# Eplin-alpha expression in human breast cancer, the impact on cellular migration and clinical outcome

**DOI:** 10.1186/1476-4598-7-71

**Published:** 2008-09-16

**Authors:** Wen G Jiang, Tracey A Martin, Jonathan M Lewis-Russell, Anthony Douglas-Jones, Lin Ye, Robert E Mansel

**Affiliations:** 1 Metastasis and Angiogenesis Research Group, Cardiff University School of Medicine, Heath Park, Cardiff CF14 4XN, UK; 2 Department of Pathology, Cardiff University School of Medicine, Cardiff, UK

## Abstract

**Introduction:**

To investigate the expression of EPLIN-α, epithelial protein lost in neoplasm, in human breast cancer tissues/cells and investigate the cellular impact of EPLIN-α on breast cancer cells.

**Experimental design:**

EPLIN-**α **was determined in tumour (n = 120) and normal mammary tissues (n = 32), and cancer cell lines (n = 16). Cell invasion, *in vitro *and *in vivo *growth of cells transfected with EPLIN-**α **were evaluated using *in vitro *invasion assay, *in vitro *and *in vivo *tumour model. Cellular migration was analysed using Electric Cell Impedance Sensing assays.

**Results:**

Low level of EPLIN-**α **was seen in tumour tissues. Grade-2/3 tumours had significantly lower levels of EPLIN-**α **compared with grade-1 (p = 0.047 and p = 0.046 vs grade-1, respectively). Patients with poor prognosis had a significantly lower levels of EPLIN-**α **compared with those with good prognosis (p = 0.0081). Patients who developed recurrence and died of breast cancer had significantly lower levels of EPLIN-**α **compared with those who remained disease free (p = 0.0003 and p = 0.0008, respectively) (median follow-up 10 years). Patients with high levels of EPLIN-**α **transcript had a longer survival than those with low levels. Over-expression of EPLIN-**α **in breast cancer cells by way of transfection rendered cells less invasive, less motile and growing at a slower pace *in vitro *and *in vivo*. An ERK inhibitor was shown to be able to abolish the effect of EPLIN expression.

**Conclusion:**

It is concluded that expression of EPLIN-**α **in breast cancer is down-regulated in breast cancer cells and tissues, a change linked to the prognosis. EPLIN-**α **acts as a potential tumour suppressor by inhibition of growth and migration of cancer cells.

## Introduction

Epithelial protein lost in neoplasm, EPLIN, was first identified as a gene that is transcriptionally down-regulated in oral cancer cells [1]. Two isoforms of EPLIN have been identified known as EPLIN-α and EPLIN-β [2], which are different by the appearance of an additional 160 amino acids at the N-terminus of the beta isoform. EPLIN is located along the actin stress fibres and focal adhesion plaques, indicating a possible role in cell morphology, migration and adhesion. Indeed, it has been recently shown to cross link and stabilise cytoskeletal filaments and promote formation of stress fibres [3], by doing so contributing to the inhibition of anchorage independent growth in transformed cells, but not in non-transformed cells [4].

EPLIN has been demonstrated to express at low levels in a range of cancer cell lines. For example, 8 out of 8 of oral cancer cell lines tested, 4 of 4 prostate cancer cells and 5 of 6 breast cancer cell lines were found to have low levels of EPLIN transcript [3,5]. Although the investigation into the biological function of EPLIN remains at preliminary stage, the information of their role in cells and the cytoskeleton and reduced expression in cancer cells indicate that the molecule may act as a tumour suppressor. However, there has been no clinical evidence to indicate as such. In a molecular screening programme, we identified EPLIN-**α **as one of the molecules whose expression was most aberrant.

The aim of the current study was to investigate the possible clinical implication of EPLIN-**α **in human breast cancer. Here, we first report that expression of EPLIN-**α **in human breast cancer is aberrant and that the levels of expression is linked to the clinical outcome of patients with breast cancer and provide evidence that by affecting cytoskeletal elements, EPLIN-**α **reduced cell growth *in vitro *and tumour growth *in vivo*. It is also a regulator of cellular migration as demonstrated by an ECIS based analysis.

## Methods

### Tissues and patients

Breast cancer cell line MDA MB-463, MDA MB-435s, MDA MB-436, MCF10A. MCF-7, ZR 7–51, MDA MB-468, BT-482, BT474, BT549, MDA MB-157, and MDA MB 231, human fibroblast cell lines IBTG3 and MRC5 were purchased from the European Collection of Animal Cell Cultures (ECACC, Salisbury, England). These breast cancer cell lines represented panel of cells with high invasiveness (MDA MB-435s, MDA MB-231), low invasiveness (MCF-7, ZR 7–51, MDA MB-463, MDA MB-436, MDA MB 468, MDA MB-157, BT549) and immortalised mammary epithelial cells (MCF-10A). Of the cell lines used, MCF-7, MCF-10A, BT-474, ZR 7–51 are known ER positive, remaining are ER negative (except BT482 and MDA MB-463 of which ER status are not clear). Most breast cancer cell lines are from ductal carcinoma which is the main type of the tumour histological types in the present study, except MDA MB-435s, MDA MB 436, MDA MB-468 which were initially described as from adenocarcinoma of the breast. Human umbilical vein endothelial cells (HUVEC) were purchased from TCS Biologicals (Oxford, England). Human endothelial cell line (HECV) was obtained from the Biology and Cellular and Molecular Pathology Dept, Naples, Italy. Breast cancer tissues (n = 120) and normal background tissues (n = 32) were collected immediately after surgery and stored in the deep freezer until use. Patients were routinely followed clinically after surgery. The median followup period was 120 months. The presence of tumour cells in the collected tissues was verified by examination of frozen sections using H & E staining by a consultant pathologist (ADJ). Clinical details of the patients are given in table 1.

**Table 1:** Clinical and pathological information of the cohort

**Groupings**	**Sample numbers**				
**Nodal status**	Node negative, n = 65		Node positive, n = 55		

**Grade**	Grade 1, n = 23	Grade 2, n = 41	Grade 3, n = 56		
**Histology**	Ductal, n = 94	Lobular, n = 14	Others, n = 12		
**TNM staging**	TNM1, n = 69	TNM2, n = 40	TNM3, n = 7	TNM4, n = 4	
**Clin.** **outcome**	Disease free, n = 81	With Metastasis, n = 7	With local recur., n = 5	Died of breast Cancer, n = 20	Died of unrelated diseases, n = 7

### Materials and regents

Rabbit anti-EPLINα antibody was from CalbioChem (Nottingham, England, UK). Cloning vector, pEF6/TOPO/his was from Invitrogen, Pasley, Scotland, UK). ROCK inhibitor was from Santa-Cruz Biotechnologies Inc., PLC-γ inhibitor, JNK inhibitor, JAK inhibitor, ERK inhibitor, MET inhibitor, Wortmannin, AG490 and Wiskostatin were from CalBiochem, Nottingham, England, UK.

### Tissue processing and extraction of RNA and generation of cDNA

Over 20 frozen successive sections from the each tissue sample were homogenised in a RNA extraction solution using a hand held homogeniser to extract total RNA. The concentration of RNA was quantified using a UV spectrophotometer. 1 μg RNA was used to generate cDNA using a commercially available RT kit (AbGene Laboratories, Essex, England).

### Detection of EPLIN-α using RT-PCR

Routine RT-PCR was carried out using a PCR master mix that was commercially available (AbGene). Primers were designed using the Beacon Designer software (version 2, Biosoft International, Palo Alto, California, USA), to amplify regions of human EPLIN-**α **that have no significant overlap with other known sequences and that the amplified products span over at least one intron, based on sequence accession number AF198455. The primers used were: 5'AAGCAAAAATGAAAACGAAG'3 and 5'ACTGAACCTGACCGTACAGACACCCACCTTAGCAATAG'3. Reactions were carried out at the following conditions: 94°C for 5 minutes, 36 cycles of 94°C for 15 seconds, 55°C for 25 seconds and 72°C for 15 seconds. PCR products were separated on a 2% agarose gel and photographed using a digital camera mounted over a UV transluminator. β-actin was used as a house keeping gene: 5'ATGATATCGCCGCGCTCG'3 and 5'CGCTCGTGTAGGATCTTCA'3.

### Quantitative analysis of EPLIN-α transcripts

The levels of EPLIN-**α **transcripts from the above-prepared cDNA was determined using a real-time quantitative PCR, based on the Amplifluor™ technology, modified from a method previous reported [6]. Briefly, pairs of PCR primers were similarly designed using the Beacon Designer software to that of conventional PCR primers, but to one of the primer, an additional sequence was added, known as the Z sequence (5'actgaacctgaccgtaca'3) which is complementary to the universal Z probe (Intergen Inc., Oxford, England, UK). A Taqman detection kit for β-actin was purchased from Perkin-Elmer. The reaction was carried out using the following: Hot-start Q-master mix (Abgene), 10 pmol of specific forward primer, 1 pmol reverse primer which has the Z sequence, 10 pmol of FAM-tagged probe (Intergen Inc.,), and cDNA from approximate 50 ng RNA. The reaction was carried out on IcyclerIQ™ (Bio-Rad, Hemel Hemstead, England, UK) which is equipped with an optic unit that allows real time detection of 96 reactions. The following condition was used in the reaction: 94°C for 12 minutes, 50 cycles of 94°C for 15 seconds, 55°C for 40 seconds (the data capture step) and 72°C for 20 seconds. The levels of the transcripts were generated from an internal standard that was simultaneously amplified with the samples. Cytokeratin-19 (CK19) was used to normalise cellularity during the analysis and primers for CK19 were 5'CAGGTCCGAGGTTACTGAC3' and 5'ACTGAACCTGACCGTACACACTTTCTGCCAGTGTGTCTTC'3, respectively [7]. Data are shown here as either the number of transcripts, or as EPLIN:CK19 ratio.

### Immunohistochemical staining of EPLIN

Frozen sections of mammary tissues (32 paired normal and 32 matched tumour tissues, as well as dissected tumour tissues) were cut at a thickness of 6 μm using a cryostat (Leica). The sections were mounted on super frost plus microscope slides, air dried and then fixed in a mixture of 50% acetone and 50% methanol for 15 minutes. Staining for each molecule was conducted on all the slides at the same time in a single batch to avoid variance in experimental conditions. The sections were then placed in "Optimax" wash buffer (a Tween 20 containing washing buffer from Sigma, Poole, Dorset, England, UK) for 5 – 10 minutes to rehydrate. Sections were incubated for 20 mins in a blocking solution that contained 10% horse serum and probed with the primary antibody (rabbit anti-human EPLIN, Calbiochem, used at dilution of 1:100, for 60 minutes). The dilution chosen here was based on the evaluation test run, during which the antibody was tested over a range of 1:10 to 1:1000. Primary antibodies were omitted in the negative controls. Following extensive washings, sections were incubated for 30 minutes in the solution containing the secondary biotinylated antibody (Multilink Swine anti-goat/mouse/rabbit immunoglobulin). Following washings, Avidin Biotin Complex (Vector Laboratories) was then applied to the sections followed by extensive washings. Diaminobenzidine chromogen (Vector Labs) was then added to the sections which were incubated in the dark for 5 minutes. Sections were then counter-stained in Gill's Haematoxylin and dehydrated in ascending grades of methanol before clearing in xylene and mounting under a cover slip.

### Construction of expression vector for human EPLIN-α

Full length human EPLIN-α cDNA was generated from a cDNA preparation of normal mammary tissues. The following primers which allowed amplification of the full length human EPLIN-**α **were used: 5'ATGGAAAATTGTCTAGGAGA'3(EPLINaExF1), 5'ATGGAAAATTGTCTAGGAGAA'3 (EPLINaExF2) and 5'TCACTCTTCATCCTCATCCTC'3 (EPLINaExR1). The reaction was carried out using high-fidelity master mix with proof reading enzymes (AbGene). Correctly amplified product was T-A cloned into a mammalian expression vector, pEF6/V5-his. The ligated products were then used to transform the TOP10 bacteria (Invitrogen) for amplification. Plasmid was extracted from *E. coli *which had correct oriented insert and purified. MDA MB 231 cells were transfected by way of electroporation using an electroporator (Easyjet, Flowgen). 48 hours after electroporation, selection medium which contained blasticidin (5 μg/ml final concentration) was used to select stably transfected strains. After verification of the expression, two stains that carried high level EPLIN-α expression were established and subsequently named as MDA-MB231^EPLINexp3^, and MDA-MB231^EPLINexp4^. Wild type and cells transfected with control plasmid respectively named MDA-MB231^wt^, MDA-MB231^pEF/His^, were used as controls during the studies.

### Electric cell-substrate impedance sensing (ECIS) based cellular motility and micromotion assays

The 9600 model of the ECIS instrument (Applied Biophysics Inc, NJ, US) were used for motility assay (wounding assay) in the study and ECIS-1600R model for wounding/cell modelling and micromotion analysis [8,9]. Cell modelling was carried out using the ECIS RbA modelling software, supplied by the manufacturer. The 8W10 arrays (8 well format with 10 probes in each well) were used in the present study. ECIS measures the interaction between cells and the substrate to which they are attached via gold-film electrodes placed on the surface of culture dishes. Following treating the array surface with a Cysteine solution, the arrays were incubated with complete medium for 1 hour. The same number of MDA-MB231^wt^, MDA-MB231^pEF/His^, MDA-MB231^EPLINexp3^, or MDA-MB231^EPLINexp4 ^(250,000 per well) in the same volume of medium (400 μl) were added to each wells. After 3 hours when confluence was reached, the monolayer was electrically wounded at 6 v for 30 seconds. Impedance and resistance of the cell layer were immediately recorded for a period of up to 20 hours. For micromotion analysis, similarly prepared cells in the array were placed into the 1600R model. Micromotion was recorded at 4000 Hz for 15 minutes. Migration and micromotion were modelled using the ECIS RbA cell modelling software.

### In vitro invasion analysis and cell growth assay

This was performed as previously reported and modified in our laboratory [10]. Briefly, transwell inserts (upper chamber) with 8 μm pore size were coated with 50 μg/insert of Matrigel and air-dried, before being rehydrated. 20,000 cells were added to each well, with or without rhHGF/SF (recombinant human hepatocyte growth factor). After 72 hours, cells that had migrated through the matrix and adhered to other side of the insert were fixed and stained with 0.5% (w/v) crystal violet. Cells that had invaded and stained with crystal violet were extracted with 10% (v/v) of acetic acid and absorbance obtained using a multiplate reader.

For cell growth assay, MDA-MB-231^WT^, MDA-MB-231^pEF6^, or MDA-MB-231^EPLINexp ^cells were plated into 96 well plate at 2,500 cells/well. Cells were fixed in 10% formaldehyde on the day of plating, and on days 1, 2, 3, 4, 5, and 6 after plating. The cells were then stained with 0.5% (w/v) crystal violet. Following washing, stained crystal was extracted with 10% (v/v) acetic acid and absorbance determined using a multiplate reader. The growth of cells are shown here as absorbance (mean ± SD).

### In vivo development of mammary tumour

Athymic nude mice (Nude CD-1) of 4–6 weeks old were purchased from Charles River Laboratories, Kent, England, UK and maintained in filter-toped units. Breast cancer cells in culture flasks were first washed using sterile BSS and treated using EDTA-Trypsin buffer. After removing EDTA-Trypsin and washing, the single cell suspension was prepared using serum free medium which also contained 0.5 mg/ml Matrigel. The cell number in the suspension is 5 × 10^6^/ml. 100 μl of this cell suspension (containing 0.5 million cells) was injected subcutaneously at the left scapula area [10]. Three groups were included: MDA MB231 wild type, MDA MB-231 control transfection, and MDA-MB-231 transfected with EPLIN-α expression constructs. Each tumour group included 6 athymic nude mice. Mice were weighed and tumour sizes measured twice weekly for 4 weeks. Mice with weight loss over 25% or tumour size larger than 1 cm in any dimension were terminated according to the UK Home Office and UKCCCR guideline. The volume of the tumour was determined using the formula: tumour volume = 0.523 × width^2 ^× length. At the conclusion of the experiment, animals were terminally anaesthetised, primary tumours were dissected, weighed and frozen at -80°C. Part of the primary tumours was fixed for histological examination.

Statistical analysis was carried out using Mann-Whitney U test and significant difference was taken at p < 0.05. Survival was analysed using Kaplan-Meier survival analysis on SPSS 12.

## Results

### Expression of EPLIN-α in human breast tissues and breast cancer cell lines

**Figure 1:**
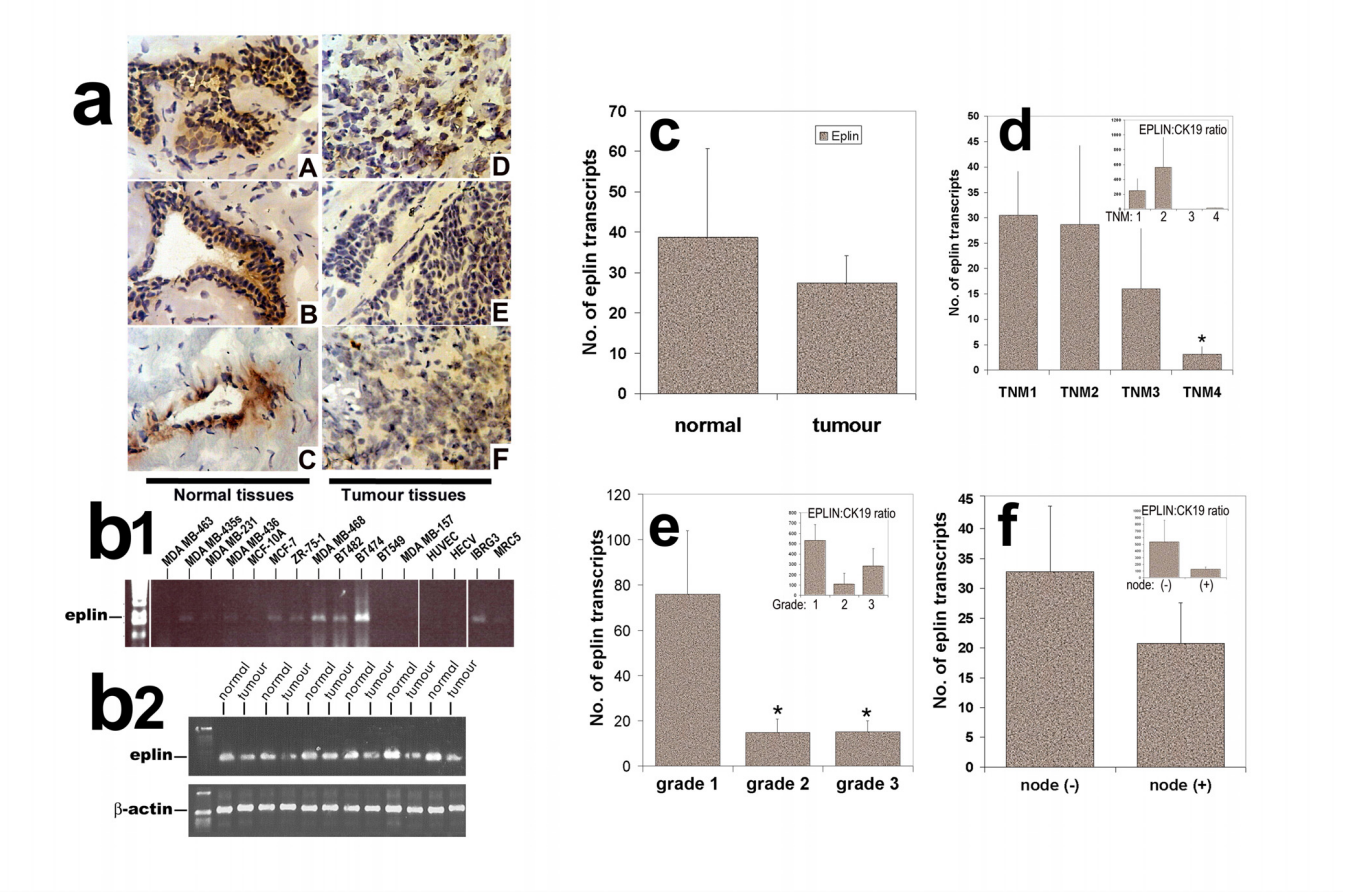
**EPLIN-**α **expression in breast cancer tissues and cell lines.**a: Immunohistochemical staining of EPLIN-**α **in normal mammary tissues (left panel) and tumour tissues (right panel) from different patients. The tumour tissues were from patients with different tumour grade. D was a grade-1 tumour (Patient ID 123), E a grade-2 tumour (ID 75a) and F grade-3 tumour (ID 113). Magnification was ×200 for the micrographs. b1: Detection of the EPLIN-**α **transcript using RT-PCR in a panel of human breast, endothelial and stromal fibroblast cell lines. b2: Detection of the EPLIN-**α **transcript in a panel of matched normal and tumour tissues. Actin was used as the housekeeping control. c: quantitative analysis of the EPLIN-**α **transcript in normal and tumour tissues. d: Levels of expression of EPLIN-**α **transcripts in breast tumour tissues in connection with TNM status (left; * p = 0.0029 vs TNM1 tumours). e: Levels of expression of EPLIN-**α **in breast tumour tissues in connection with grade (left, * p < 0.05 vs grade 1 tumours) and F: with nodal status (right). Inserts in d, e and f are values of EPLIN-**α **transcript that have been normalised by CK19 (shown as EPLIN:CK19 ratio).

We first analysed the expression pattern of EPLIN-α in paired breast tissues and breast cancer cell lines. EPLIN-**α **protein was found in the cytoplasmic region of normal mammary epithelial cells. Stromal cells had very little staining (figure 1a). This would indicate, to some degree, that EPLIN-**α **is primarily located epithelial cells. However, in tumour tissues, EPLIN-**α **staining in cancer cells was substantially weaker than in normal epithelial cells (figure 1a). The pattern is largely supported by the analysis of the EPLIN-**α **transcript using conventional PCR (figure 1b2). Tumour tissues did indeed show a weak signal compared with normal tissues. Most breast cancer cell lines showed a weak presence of EPLIN-**α **transcript. BT474 and MDA MB-468 showed a stronger signal of the transcript (figure 1b1). Interestingly, two of the fibroblast cell lines also showed a weak signal for EPLIN-α. Interestingly, a human endothelial cells, HUVEC and HECV, had little EPLIN-**α **transcript.

We went on to quantify the levels of EPLIN-**α **transcript in breast tumour tissues. Although the levels of EPLIN-**α **in breast cancer tissues was lower (27.4 ± 6.8 copies per 50 ng RNA) compared with normal tissues (38.8 ± 22), the difference is not statistically significant (p = 0.06), primarily due to the high level of variance seen in normal tissues (Figure 1c). When EPLIN-**α **transcripts were normalised by CK19, the EPLIN:CK19 ratio was 2888 ± 2412 in normal and 329 ± 167 in tumour tissues (p = 0.0495).

We further analysed the levels of EPLIN-**α **transcript in connection with the grade of breast tumours. Figure 1e has shown that grade 2 and grade 3 tumour had significantly lower levels of EPLIN-**α **compared with grade 1 breast tumours (p = 0.047 and p = 0.046 vs grade 1, respectively, insert – matched EPLIN:CK19 ratio). Furthermore, the relationship between EPLIN-**α **and TNM status was also analysed. Figure 1d3 has clearly shown that there appeared to be a stepwise decrease of levels of EPLIN-**α **from TNM1 to TNM4 tumours. However, significant difference was only seen in TNM4 tumours when compared with TNM1 tumours (p = 0.0029) (figure 1d). Although node positive tumours had lower levels of EPLIN-**α **transcript (20.8 ± 6.8) when compared with node negative tumours (32.8 ± 6.8), the difference was nonetheless insignificant (figure 1f). We also compared the levels of EPLIN-**α **in the main tumour types in the cohort. The levels were seen similar between ductal and lobular tumours (22.7 ± 6.8 vs 29.5 ± 20, p > 0.05).

### Expression of EPLIN-α in breast tumour is correlated with prognosis

**Figure 2:**
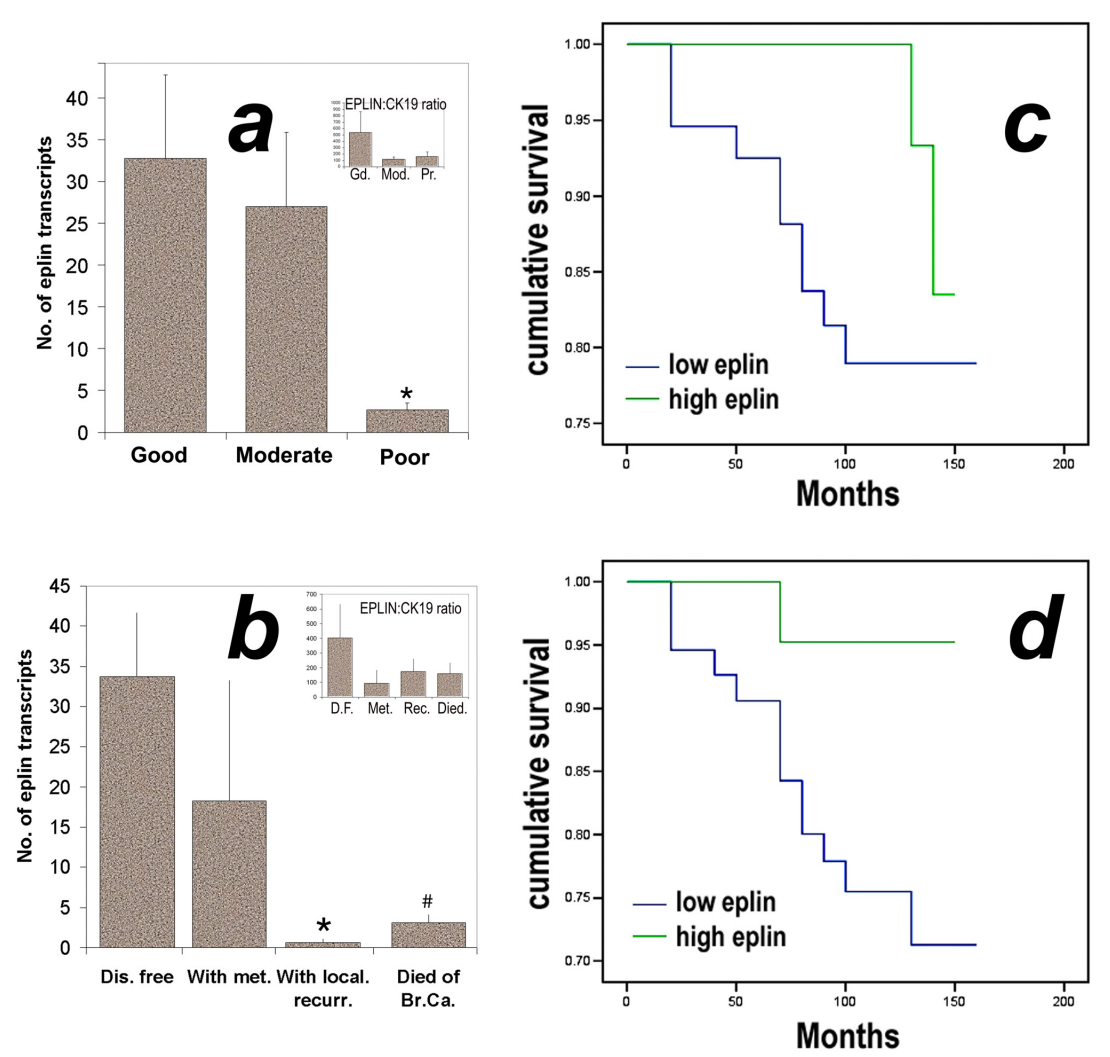
**a: EPLIN-**α **and its correlation with predicted prognosis (left) and clinical outcome (right).** The predicted prognosis was based on the NPI value of each patient and that good, moderate and poor prognosis had NPI value either < 3.4, 3.4–5.4 or > 5.4. * p = 0.0081 vs good prognostic group. b. Levels of EPLIN-**α **transcript and clinical outcome. * p = 0.0003, # p = 0.0008 vs disease free patients. c and d: levels of EPLIN-**α **transcript and overall survival (c) and disease free survival (d). Patients with high levels of EPLIN-**α **transcript had a significantly longer overall (p = 0.0461, in c) and disease-free survival (p = 0.0345, in d) than those with low levels of the transcript. Inserts in a and b are values of EPLIN-**α **transcript that have been normalised by CK19 (shown as EPLIN:CK19 ratio).

We have used the Nottingham Prognostic Index (NPI) as an indicator to assess the relationship between EPLIN-**α **transcript and a predicted prognosis. Patients were divided into three groups, good, moderate and poor prognosis, based on the NPI values. It was found that patients with poor prognostic index had the lowest levels of EPLIN-**α **(figure 2 left) amongst three groups. The difference between patients with poor prognosis and good prognosis was highly significant (p = 0.0081) (figure 2a).

### EPLIN-α expression is correlated with clinical outcome in patients with breast cancer

The current cohort has a 120 month follow up. Based on the clinical outcome at the final followup, patients were divided into those who remained disease free, with metastatic diseases, with local recurrence, and those who died of breast cancer (patients died of causes unrelated to breast cancer was excluded from the current analysis). As can be seen from figure 2b, patients who remained disease had the highest levels of EPLIN-**α **amongst all the patients. It is interesting to note that patients who developed local recurrence and who died of breast cancer exhibited significantly lower levels compared with those who remained disease free (p = 0.0003 and p = 0.0008, respectively).

A survival analysis using Kaplan-Meier method has shown that patients with low EPLIN-**α **tumours had a shorter overall survival (135.3 (123.6–147.1, 95%CI) months) compared with those with high EPLIN-**α **tumours (141.7 (138.5–148.7) months, p = 0.0461) (figure 2c). Similarly, low levels of EPLIN-**α **was associated with shorter disease free survival (129.4 (116.3–142.2) months) compared with high levels of EPLIN-**α **(140 (132.4–147.7) months), p = 0.0345 (figure 2d).

### Breast cancer cells over-expressing EPLIN-α were less invasive and had a slower pace of growth in vitro and in vivo

**Figure 3:**
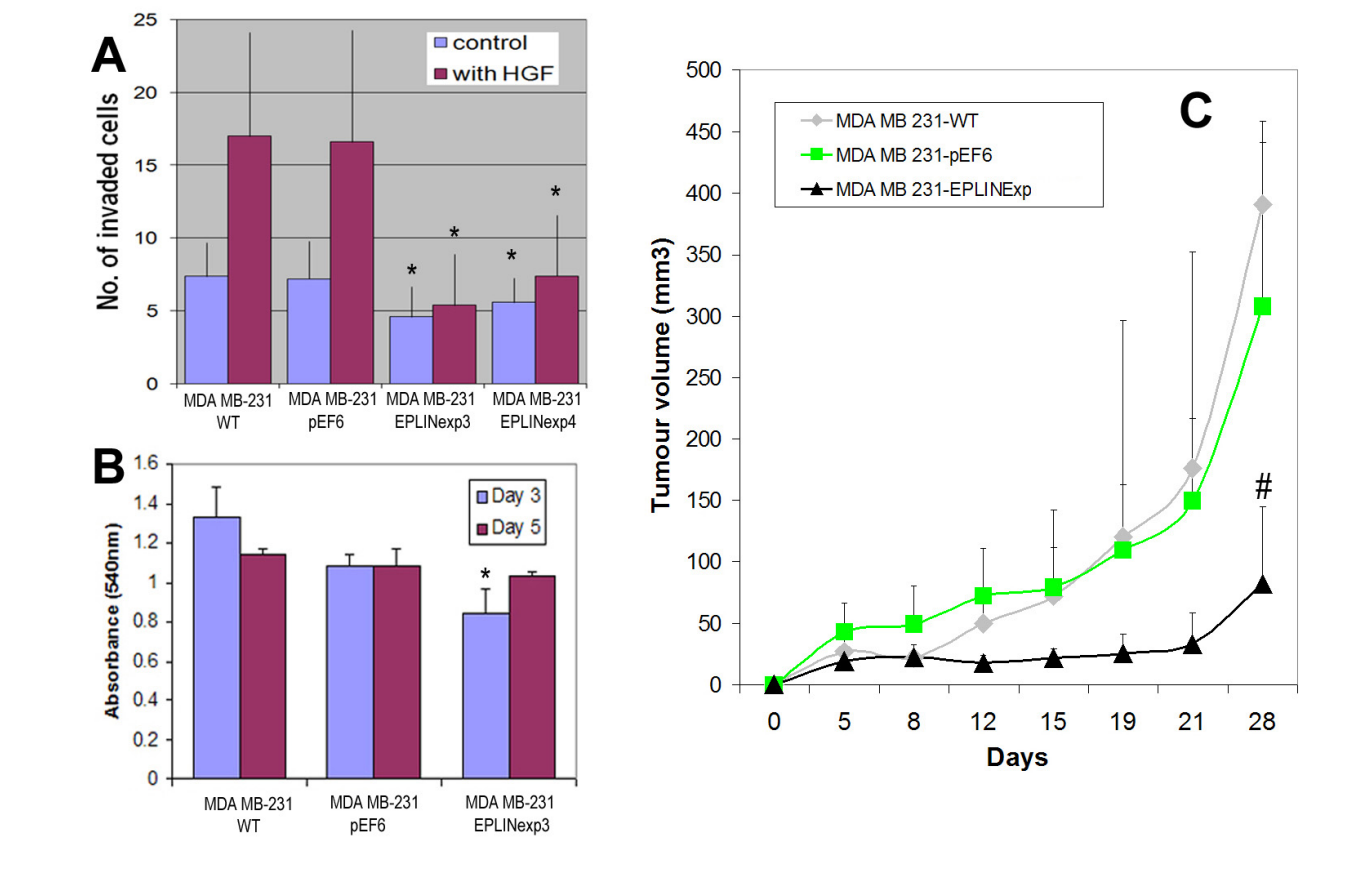
**EPLIN-**α **expression and impact on *in vitro *invasion(A), *in vitro *growth (B) and *in vivo *tumour growth (C) of breast cancer cells.** * p < 0.05 vs respective controls; # p < 0.001 vs control wild type and control groups.

Using a matrigel based *in vitro *invasion assay, it was shown that MDA MB231 cells which over-expressed EPLIN-α by way of transfection had significantly reduced invasiveness compared with both wild type and control transfected cells (figure 3A). *In vitro *cell growth assay showed a significant lower rate of growth of EPLIN-**α **transfected breast cancer cells (Figure 3B).

In an athymic nude mice model, it was shown that EPLIN-**α **transfected breast cancer cells grew at a significantly slower pace compared with control cells. The difference of tumour size was seen from early time points (after 8 days), and the overall difference between EPLIN-**α **transfected and wild type or transfection control both were highly significant (p < 0.001 by two-way ANOWA) (figure 3C).

### Electric cell impedance sensing (ECIS) based cell motility assay

**Figure 4:**
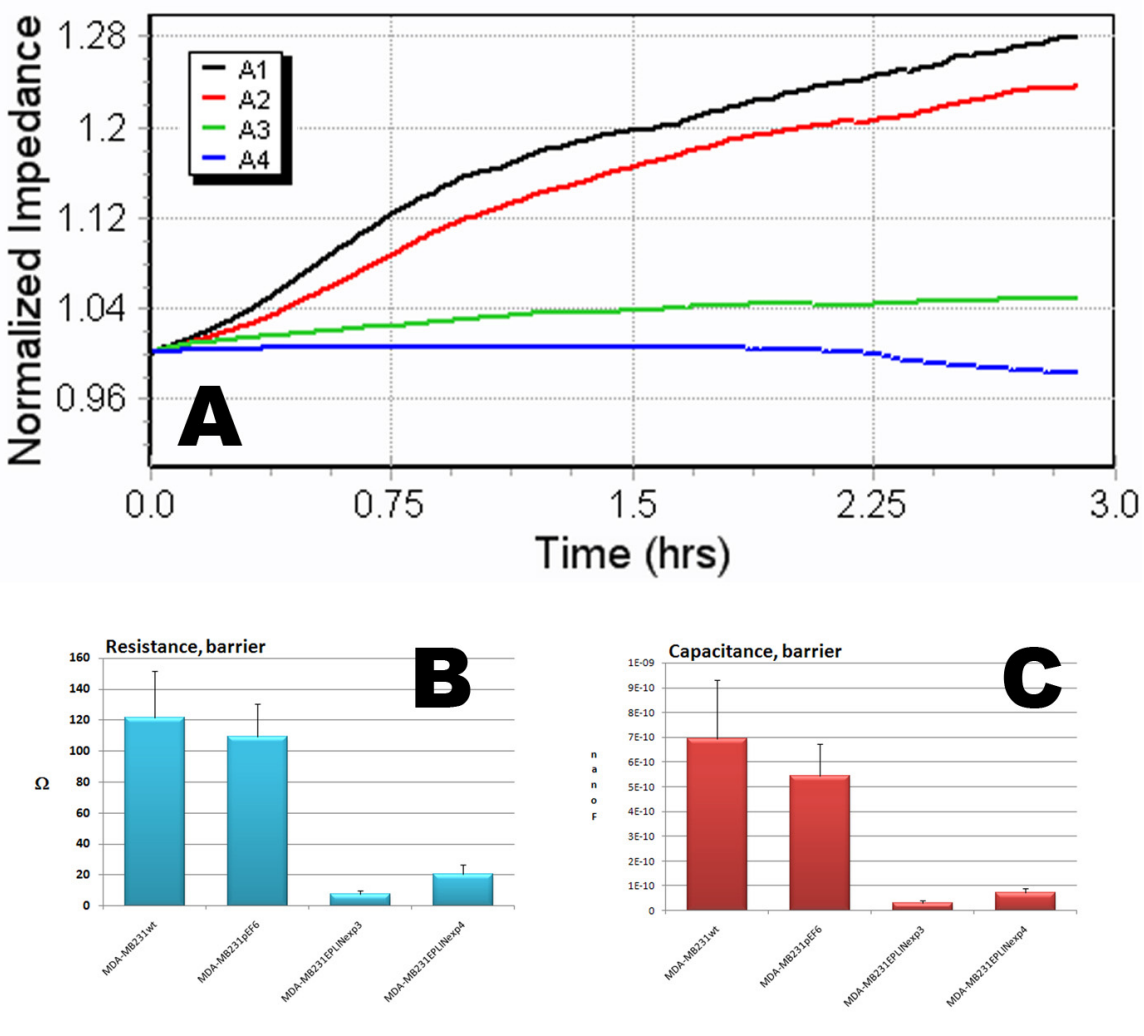
**EPLIN-**α **expression on cell migration as analysed by ECIS.** A: wounding assays. Cells were first wounded at 6 v for 30 sec. The impedance change was shown. Electrode – A1: MDA-MB231^wt^, A2: MDA-MB231^pEF/His^, A3: MDA-MB231^EPLINexp3^, A4: MDA-MB231^EPLINexp4^. The breast cancer cells which over-expressed EPLIN-**α **showed a markedly reduced migration. B and C: ECIS RbA modelling of cell motility indicated a significant reduction of motility in EPLIN-**α **transfected cells as shown by resistance in B and capacitance in C. * p < 0.01 vs MDA-MB231^wt ^and MDA-MB231^pEF/His^.

Here, we adopted the Electric Cell Impedance Sensing (ECIS) method to investigate the impact of EPLIN-**α **over-expression on cell motility. As shown in Figure 4A, EPLIN-**α **over-expressing breast cancer cells showed a dramatic slowdown in recovery after electric wounding. Using ECIS-RbA modelling system, it was shown that EPLIN-**α **over-expression in the MDAMB231^EPLINexp ^cells resulted in a significant reduction in both resistance and capacitance, when compared with both the wild type cells and the control cells (figure 4B and 4C).

**Figure 5:**
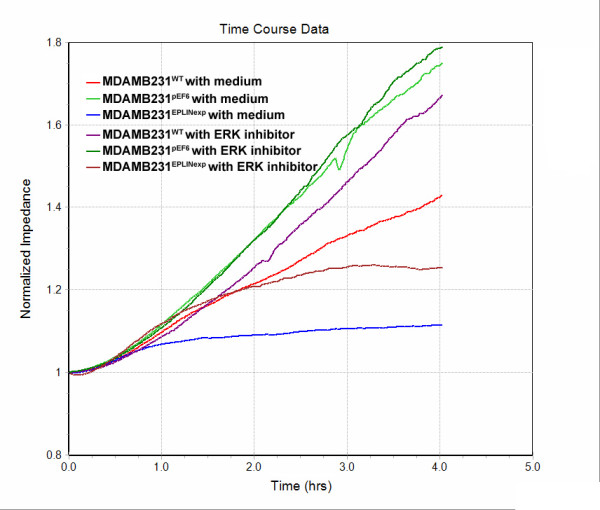
**ERK inhibitor reversed the action of EPLIN-**α **, an ECIS based wounding analysis.** Wild type, control cells and EPLIN-**α **expression MDA MB231 cells were tested in the absence or presence of ERK inhibitor. The reduction of migration in MDA MB231 over-expressing EPLIN-**α **was reverse by ERK inhibitor.

In searching for the potential pathway(s) that may be responsible for the action of EPLIN-**α **, we have screened a panel of small molecule inhibitors to some of the key signalling pathways that are linked to cell motility. They included ROCK inhibitor, JAK3 inhibitor, JNK inhibitor, PI3K inhibitor, PKC inhibitor and ERK inhibitor. Only the ERK inhibitor was seen to partially restore the inhibitory effect of EPLIN-**α **on the motility of the cancer cells as shown in figure 5. ERK inhibitor has only a marginal effect on the motility of the control breast cancer cells, however, it reverse the inhibition of motility in the MDA MB231^EPLINexp ^cells to nearing the control level. This was more obvious at the early phase (within 2 hours after wounding) (figure 5).

## Discussion

In the current study, we have shown that EPLIN-**α **, an epithelial protein frequently lost in cancer cells, is also aberrantly expressed in clinical breast tumour tissues. We also report that the levels of EPLIN-**α **are correlated with the clinical outcomes and long term survival of the patients with breast cancer. The study further demonstrates that expression of EPLIN-**α **linked to the reduction of growth of breast cancer cells *in vitro *and *in vivo*, and inhibition of cellular migration via an ERK dependent pathway.

EPLIN has been found to be transcriptionally expressed at lower levels in a limited number of tumour cells, including breast cancer cells [1-3]. This is clearly seen in the present study in which only few of the breast cancer cell lines expressed the gene transcript. Perhaps the most important observation seen in the present study is the link between the level of EPLIN expression and the clinical outcome. An inverse correlation was seen between the level of the EPLIN transcripts and tumour grade, nodal status and tumour staging. A highly significant link was seen between low levels of expression and a poor clinical outcome and shorter disease free and overall survival. These data clearly indicate that EPLIN may act as a potential prognostic indicator and that the molecule may act as a protective factor in patients with breast cancer. Information of the clinical relevant of EPLIN in human cancers in the literature of any type is limited. In another recent study, there has been indication that EPLIN transcript may also lost in colorectal cancer tissues [11]. The present study is the first to demonstrate a clear clinical relevance between EPLIN-**α **and clinical outcome.

This important clinical link has potential cellular explanations, as demonstrated in the present study and indeed in the literature. EPLIN-**α **protein was shown to be an actin cross linking protein that bundles actin in the cells and stabilises the cytoskeletal filaments. By doing so, EPLIN protein inhibits cell motility [5,12]. In the present study, we have also shown that forced expression of EPLIN-**α **in the breast cancer cell line, MDA MB-231 which is negative in EPLIN-**α **expression, resulted in the cells to be less aggressive, namely with reduced migration, invasion and *in vitro *growth. The EPLIN-**α **expressing cells also displayed a significantly reduced rate of growth *in vivo*. Collectively, the present study shows that EPLIN, a potential cell migration regulating protein, is inversely associated with the aggressiveness and clinical outcome of human breast cancers. This is likely via its inhibitory role on cell growth and migration.

Although EPLIN has been shown to be an actin bundling protein, the precise mechanism is yet to be fully established. It has been shown that EPLIN inhibits the ARP2 mediated nucleation of actin filaments. During the preparation of the manuscript, two reports have shown that EPLIN also mediates the linkage between the Cadherin/catenin complex and F actin [13] and that one potential pathway in these links is the extracellular signal-regulated kinase (ERK) pathway [14]. These recent findings support the present study which demonstrates that inhibition of ERK pathway resulted in reversal of EPLIN mediated reduction of cellular migration. This is interesting, as ERK has becoming a therapeutic target in recent years.

In conclusion, EPLIN-**α **is a powerful regulator of the cellular motility of breast cancer cells. Breast cancer cells expressing EPLIN-**α **are less motile and grow slowly in *vivo*. Together with the clinical relevance as demonstrated in the present study, EPLIN-**α **is an important prognostic indicator and may be an important target when considering therapies. We are currently examining the molecular pathways involved in EPLIN-**α **mediated cell migration.

## Competing interests

The authors declare that they have no competing interests.

## Authors' contributions

WGJ contributed study design, experimental work, manuscript preparation; TAM, LY and JMLR contributed to experimental work; ADJ and REM contributed to sample collection and pathological evaluation.
